# Clinical Reflections

**Published:** 1885-07

**Authors:** Ad. Lippe

**Affiliations:** Phila.


### CLINICAL REFLECTIONS.

AD. LIPPE, M. D., PHILA.

A very corpulent lady, fifty years of age, had for several years suffered from dysentery always at the end of the summer, and had been relieved promptly by a few doses of Kali bichro. In the summer of 1884 the dysentery returned at an earlier date then before, and the Kali bichro., which was given her to be taken on such a return, did not relieve her; she grew much worse and came to town for treatment. She was quite comfortable in the morning, seldom had any pain after breakfast, and very rarely an evacuation before dinner. As soon as she dined the violent, cutting pain in the intestines began, and sometimes compelled her to leave the table; she would then pass, with much pain and straining, very thin brown fæces with mucus and flatulence; the pain was not relieved by the evacuation, the pressure continued, and the great soreness of the abdomen increased; from four to eight evacuations followed and ceased after midnight, when the pains gradually diminished. She had very little appetite, and dreaded to eat on account of the very distressing pains; had much thirst, but did not drink much for fear of pains. Lycopodium, Sulphur, and Carbo veg. afforded no relief. Thrombidium finally was given, requiring a repetition every three to six days till the bowels became entirely relieved; the improvement, though slow, was permanent, and nothing of the kind has returned again.

Last January a gentleman of full habits, subject to attacks of rheumatism and congestion of the liver, complained that whenever he began to eat his dinner at five P. M. he experienced violent pain in the intestines, and this pain increased till he had to seek relief very suddenly; he then passed very thin fæces and some mucus with some flatulence and great tenesmus; the pains were not relieved till he had three to four similar passages, when he ceased to suffer till he dined again next day. After he had so suffered for three days he asked for medicine. One dose of Thrombidium completely cured him in a few days.

*Comments:* Thrombidium will not often be indicated in dysenteric attacks, as the indications above described are very infrequent. Each drug has its own peculiar sick-making characteristics, and, therefore, curative effect on the living organism. The aim of the progressive healing art is unquestionably to ascertain to a positive certainty what these peculiar and characteristic properties of each drug are. The only possible means of ascertaining these properties is to prove the drug first, and by the clinical experiment ascertain the correctness of the provings, and finally to find the peculiar and characteristic symptoms of that drug, symptoms peculiar to it and to no other drug. In this instance we find that Dr. Bell, in his *Therapeutics of Diarrhœa*, calls attention to Thrombidium. The two cases related above show an aggravation in the afternoon, but this does not prove the morning aggravation observed by others to be incorrect. There are in these two cases as well as in a few cases previously cured by Thrombidium some peculiar characteristic symptoms. The abdominal distress begins while eating (dinner), is not relieved by the evacuations, which are unceasing, and as long as they continue are accompanied by tenesmus. The discharges are very thin fæces mixed with mucus, and cause flatus to pass without relief. Here we find characteristic symptoms the absolute reverse of the conditions we find under Gambogia. Both remedies have similar symptoms before the stool, have very similar evacuations, but Gambogia has great relief from the abdominal pains and distress after the evacuation, while Thrombidium has no relief whatever after an evacuation. Croton tiglium has aggravation after drinking and eating just like Thrombidium, but otherwise differs in the character of the stools and in their sudden, violent expulsion. Thrombidium causes straining, tenesmus, painful, slow expulsion of different stools. Although Theridion has been proven—the provers' day-books have been published in full in the *Hahnemannian Monthly*, Vol. I—it is strange that we have had so very few clinical reports of cures from it. When we meet such rare cases, and by diligently searching for the similar remedy find it and cure the sick, the conviction must become strong that we need more provings to meet all the possible, ever-changing conditions of sickness; that, above all things, we stand in need of more knowledge of the peculiar characteristic symptoms of our remedies. We also become convinced that it is absolutely necessary to "*individualize*," and that in that manner only can we entertain the hope to make medicine a positive science. The modern generalizer, who is just proudly satisfied to have scientifically diagnosticated "dysentery," will boldly and unscientifically seek for a specific for that disease; he will, as of old, unsuccessfully battle with the disease, and then resort to "palliatives" because he failed to apply the only possible law of cure correctly. And now comes the next question to be solved—How do we, with absolute certainty, obtain the peculiar characteristic symptoms of the various drugs? They lay concealed in the day-books of the provers. The ever-active progressionist has arranged these day-books in such manner as makes them useful and accessible to the practitioner; he has brought them into a systematic order so as to enable the physician to find how every organ is affected and under what conditions these effects are produced; still further to facilitate the researches of the busy practitioner, Repertories were made. The physician anxiously seeking for a similar remedy finds it a much easier task after using a good Repertory than will the possessor of the latest fashion—a work called "*A Cyclopædia of Drug Pathogenesy*." The Repertory alone will never clearly show the most similar remedy. If there is still a doubt remaining as to the next similar remedy, the day-books of the provers should be examined. All of this is a laborious work, but the reward soon follows, the highest reward the healer can ever expect to receive—the curing of the sick and an addition to our "Drug Pathogenesy." There are others who, in their desire to progress backward and to be recognized by the Regulars as one of them, spurn hard work, and in their zeal for recognition blab about generalization as did a celebrated English friend whom we have reported on page 303 of THE HOMŒOPATHIC PHYSICIAN, Vol. I, who proclaimed that "Asthenopia is the morbid, ocular condition here indicated as the sphere of Ruta." And now we are invited to resort to the reading of the day-books of provers in every case, and that by a learned man who could not find anything under Ruta save "Asthenopia." Shortsighted is the mover of such a sifting process of our glorious materia medica, to be sure. Let us, nevertheless, not be discouraged by such silly blabbing, but continue to exert ourselves to find the peculiar characteristic symptom of every drug and thereby advance the healing art.

